# RNA-targeted therapy corrects neuronal deficits in PACS1 syndrome mice

**DOI:** 10.21203/rs.3.rs-2440581/v1

**Published:** 2023-01-27

**Authors:** Sabrina Villar-Pazos, Laurel Thomas, Yunhan Yang, Kun Chen, Jenea B. Lyles, Bradley J. Deitch, Joseph Ochaba, Karen Ling, Berit Powers, Sebastien Gingras, Holly B. Kordasiewicz, Melanie J. Grubisha, Yanhua H. Huang, Gary Thomas

**Affiliations:** 1 Department of Microbiology and Molecular Genetics, University of Pittsburgh School of Medicine, Pittsburgh, PA 15219, USA; 2 Ionis Pharmaceuticals, Carlsbad, CA, USA; 3 Department of Immunology, University of Pittsburgh School of Medicine, Pittsburgh, PA 15213; 4 Department of Psychiatry, University of Pittsburgh School of Medicine, Pittsburgh, PA, USA; 5 Translational Neuroscience Program, University of Pittsburgh School of Medicine, Pittsburgh, PA, USA

## Abstract

Neurodevelopmental disorders (NDDs) are frequently associated with dendritic abnormalities in pyramidal neurons that affect arbor complexity, spine density, and synaptic communication ^1,2^. The underlying genetic causes are often complex, obscuring the molecular pathways that drive these disorders ^3^. Next-generation sequencing has identified recurrent *de novo* missense mutations in a handful of genes associated with NDDs, offering a unique opportunity to decipher the molecular pathways ^4^. One such gene is *PACS1*, which encodes the multi-functional trafficking protein PACS1 (or PACS-1); a single recurrent *de novo* missense mutation, c607C>T (PACS1^R203W^), causes developmental delay and intellectual disability (ID) ^5,6^. The processes by which PACS1^R203W^ causes PACS1 syndrome are unknown, and there is no curative treatment. We show that PACS1^R203W^ increases the interaction between PACS1 and the α-tubulin deacetylase HDAC6, elevating enzyme activity and appropriating control of its posttranscriptional regulation. Consequently, PACS1^R203W^ reduces acetylation of α-tubulin and cortactin, causing the Golgi to fragment and enter developing neurites, leading to increased dendrite arborization. The dendrites, however, are beset with diminished spine density and fewer functional synapses, characteristic of ID pathology. Treatment of PACS1 syndrome mice with PACS1- or HDAC6-targeting antisense oligonucleotides restores neuronal structure and synaptic transmission, suggesting PACS1^R203W^/HDAC6 may be targeted for treating PACS1 syndrome neuropathology.

Confocal analysis of fibroblasts isolated from PACS1 syndrome patients and their healthy parents (control) revealed PACS1^R203W^ profoundly disturbed Golgi positioning and microtubule (MT) organization (Figs. 1a and S1a). In patient cells, the Golgi fragmented into dispersed mini-stacks, whereas in control cells the Golgi ribbon characteristically collected in the paranuclear region. In addition, MTs in patient cells were disorganized and failed to emanate from a single microtubule organizing center (MTOC), as observed in control cells. Consistent with this observation, a MT regrowth assay revealed patient cells contained multiple MTOCs, whereas control cells, as expected, contained a single paranuclear MTOC (Fig. S1b).

Golgi positioning and MTOC organization are critically dependent upon the acetylation state of α-tubulin at Lys^40^. In fibroblasts, Ac-Lys^40^-α-tubulin stabilizes MTs, enabling the Golgi ribbon to collect over the paranuclear MTOC ^7^. By contrast, α-tubulin deacetylation destabilizes MTs, causing dissolution of the MTOC and dispersal of Golgi mini-stacks. Consistent with this model, Western blot analysis revealed that the level of Ac-Lys^40^-α-tubulin is reduced in PACS1^R203W^ patient cells (Figs. 1b and S1c). To determine whether the reduced level of Ac-α-tubulin contributes to the Golgi fragmentation, patient cells expressing K^40^Q-α-tubulin, an acetylated tubulin mimic, or non-acetylatable K^40^R-α-tubulin were analyzed by confocal microscopy (Fig. 1c). Golgi positioning was rescued by K^40^Q-α-tubulin but not K^40^R-α-tubulin, suggesting the reduced level of Ac-Lys^40^-α-tubulin in PACS1 syndrome patient cells causes both the Golgi fragmentation and disorganized MTs.

Acetylation of α-tubulin is regulated by the class II lysine deacetylase HDAC6 and the class III enzyme SIRT2 ^8^. Co-immunoprecipitation (co-IP) studies showed that the R203W mutation increased the interaction between PACS1 and HDAC6 but had no effect on the interaction with SIRT2 (Figs. 1d and e). Next, PACS1^R203W^ fibroblasts and their parental controls were treated with selective inhibitors of HDAC6 or SIRT2 activity ^9^. Only inhibition of HDAC6 rescued Golgi positioning (Fig. S2a). The importance of PACS1^R203W^ and HDAC6 to the Golgi fragmentation was confirmed with siRNA knockdown. Depletion of either protein restored Golgi positioning in the PACS1^R203W^ patient cells (Figs. 1f and S2b).

The physiologic interaction between PACS1 and HDAC6 was established by co-IP of the endogenous proteins (Fig. 1g). We next investigated how PACS1^R203W^ and HDAC6 interact to affect enzyme activity. Of the 18 HDACs, only HDAC6 possesses two catalytic domains, CD1 and CD2 (Fig. 1h). CD2 accounts for the protein’s physiological tubulin deacetylation activity whereas the biological function of CD1 is largely unknown ^10^. Co-IP between PACS1 and a battery of HDAC6 deletion mutants showed that PACS1 interacts with the HDAC6 CD1 region. A reciprocal mapping strategy showed HDAC6 interacts with the PACS1 FBR, which harbors the disease-causing R203W change and binds numerous client proteins as well as trafficking adaptors (Fig. 1i) ^6,11^. In addition, HDAC6 interacts with the PACS1 CTR, a region of unknown function but contains the sequence that defines the PACS-1 family (Pfam #PF10254). Surprisingly, the R203W change reduced the interaction between HDAC6 and a truncated PACS1 containing only the FBR (Fig. S2c), suggesting the mutation allosterically increases the interaction between HDAC6 and full-length PACS1^R203W^ (see panel 1D). The effect of PACS1^R203W^ on HDAC6 activity was assessed in an *in vitro* assay, following isolation of HDAC6 from cells expressing HDAC6 alone or together with PACS1 or PACS1^R203W^ (Fig. 1j). HDAC6 activity was greater following co-expression with PACS1^R203W^ compared to PACS1, consistent with the reduced acetylation of α-tubulin in patient cells. Together, these findings suggest the R203W mutation increases the multi-domain interaction between PACS1^R203W^ and HDAC6 to aberrantly potentiate deacetylase activity.

To assess the impact of PACS1^R203W^ *in vivo*, we used CRISPR/Cas9 gene editing to knock-in Cre-inducible, HA-tagged human PACS1 or PACS1^R203W^ at the murine Rosa26 safe harbor locus ^12^ (Fig. S3a). The resulting R26^P1^ and R26^P1R203W^ lines were crossed with *Emx1*^*Cre*^ mice to induce cassette expression in excitatory pyramidal neurons of the hippocampus and cerebral cortex, a key site of autism/ID pathophysiology ^2,13^. Western blot and immunohistochemical staining showed *Emx1*^*Cre*^ faithfully induced expression of HA-tagged PACS1 or PACS1^R203W^ at levels similar to endogenous PACS1 in the cortex and hippocampus (Figs. 2a and b). As premature activation of HDAC6 can interfere with radial migration of cortical neurons ^14^, coronal sections were also stained for SATB2 (upper layer marker) and CTIP2 (BCL11B, layer 5 marker), revealing that *Emx1*^*Cre*^-induced PACS1^R203W^ does not appear to disturb cortical layering (Fig. S3b).

A close examination of the CA1 neurons in the R26 mice revealed that PACS1 concentrated in the cell body whereas PACS1^R203W^ appeared to redistribute along the apical dendrites (Fig. 2c). In developing neurons, the Golgi ribbon disassembles into mini-stacks, which migrate into the growing dendrites to support membrane traffic and possibly serve as secondary MTOCs ^15^. As PACS1^R203W^ triggers Golgi fragmentation (Fig. 1), we analyzed the effect of the R203W mutation on Golgi positioning and dendrite morphology in dissociated CA1 hippocampal neurons. In R26^P1^ neurons, the Golgi remained in the cell body together with PACS1, as observed *in vivo* (Figs. 2d and S3c). By contrast, in R26^P1R203W^ neurons, the Golgi fragmented and deployed to the base of the primary neurite and collected, with PACS1^R203W^, in varicosities along the developing axon and dendrites, which were also longer than those of R26^P1^ neurons.

We next evaluated the impact of PACS1^R203W^/HDAC6 on dendrite arborization *in vivo*. Specifically, R26^P1^ and R26^P1R203W^ mice were co-injected at post-natal day 1 (P1) with Cre-inducible AAV-FLEX-tdTomato, to space fill neurons, together with an HDAC6-specific antisense oligonucleotide (H6ASO) or a negative control ASO (nASO) (Fig. 2e). A single injection of the H6ASO was found to effectively and durably deplete cerebral *Hdac6* mRNA and protein and correspondingly increase the level of Ac-Lys^40^-α-tubulin (Figs. 2e and S3d). Sholl analysis of the tdTomato^+^ CA1 neurons at P18 revealed that PACS1^R203W^ increased dendritic arbor complexity, both in dendrite length and the number of branch points (Fig. 2f). Importantly, the H6ASO reversed the overbranching, resulting in a dendritic arbor indistinguishable from that detected in PACS1 mice.

Examination of the tdTomato^+^ secondary apical dendrites revealed PACS1^R203W^ markedly reduced spine density and induced accumulation of varicosities, characteristic of ID and neurodegenerative disorders (Fig. 3a) ^16^. The H6ASO fully restored spine density to levels observed in PACS1 mice, suggesting the PACS1^R203W^/HDAC6 complex functions in dendritic spines. In support of this possibility, confocal analysis of DIV18 hippocampal neurons revealed a pronounced co-localization of HA-tagged PACS1 or PACS1^R203W^ with the glutamatergic spine maker, PSD95 (Fig. 3b). In addition, endogenous PACS1 and HDAC6 were found to fractionate with synaptosomes and to co-localize in PSD95^+^ dendritic spines (Figs. 3c and S4a), consistent with a report that the *Pacs1* mRNA belongs to a small subset of neuronal transcripts that localize to hippocampal synapses ^17^. Notably, PACS1^R203W^ reduced the level of acetylated cortactin (Ac-cortactin), which has a structural role in synapses and is an HDAC6 substrate (Fig. 3d) ^18^. As Ac-cortactin stabilizes the PSD95 scaffold that organizes cell-surface AMPA receptors (AMPARs) ^19^, these findings suggest PACS1^R203W^ and HDAC6 traffic to dendritic spines where they trigger excessive deacetylation of cortactin to disturb synapse organization.

The lower spine density in R26^P1R203W^ mice suggested that the R203W mutation reduces the number of functional synapses. To test this possibility, brain slices were prepared from juvenile R26^P1^ or R26^P1R203W^ mice that had been treated on P1 with H6ASO or nASO (Fig. 2e), and miniature postsynaptic current (mPSCs) were recorded from L2/3 pyramidal neurons within the medial prefrontal cortex (mPFC) ^20^. PACS1^R203W^ markedly reduced AMPAR-mediated glutamatergic excitatory mPSC (mEPSC) amplitude and frequency but had no effect on GABAergic inhibitory mPSCs (mIPSCs, Figs. 3e and S4b). These findings are consistent with earlier reports that HDAC6-dependent deacetylation of presynaptic α-tubulin reduces mEPSC frequency whereas deacetylated cortactin reduces clustering of the PSD95 postsynaptic scaffold, which, in turn, suppresses mEPSC amplitudes ^18,19^. Importantly, the H6ASO treatment in R26^P1R203W^ mice fully restored mEPSC amplitude and frequency to levels observed in R26^P1^ mice, which were unaffected by HDAC6 depletion (Fig. 3e). The reduced mEPSCs implies a broader impact of PACS1^R203W^/HDAC6 on neuronal communication, considering that mEPSCs are not only a measure of functional synapses, but also modulate action potential firing and influence the maturation and stability of synaptic networks; reductions in mEPSCs in ID interfere with local translation ^21,22^. Thus, the ability of the H6ASO to restore mEPSCs in R26^P1R203W^ mice suggests targeting HDAC6 may significantly benefit synaptic networks in PACS1 syndrome.

The R26 knock-in strategy, while accelerating our ability to identify a key molecular pathway underpinning PACS1 syndrome, prevented testing whether PACS1^R203W^ itself can be safely and effectively targeted with ASOs, which would eliminate all molecular pathways disturbed by the disease mutation. We, therefore, generated PACS1-deficient mice (Pacs1^Δ4bp/Δ4bp^ (Pacs1^KO^)) to determine whether loss of *Pacs1* affects viability (Fig. S5a). Mating of heterozygous Pacs1^+/Δ4bp^ (Pacs1^HET^) mice demonstrated that loss of *Pacs1* reduced survival at weaning, and the surviving pups were smaller than their WT littermates but were viable and fertile (Table S1 and Fig. S5b). Western blot analysis revealed that loss of *Pacs1* increased the levels of Ac-cortactin in brain (Fig. S5c). Similarly, the level of Ac-Lys^40^-α-tubulin was higher in Pacs1^KO^ embryonic fibroblasts (MEFs) than in WT MEFs (Fig. S5d). Interestingly, loss of *Pacs1* was coupled to an increased expression of its paralogue, *Pacs2*, both in the brain and in MEFs, suggesting PACS2 buffers an essential function of PACS1 (Figs. S5d and e). In support of this possibility, crossing Pacs1^HET^;Pacs2^HET^ double heterozygotes failed to produce double knockout pups (Table S2). Next, the effect of *Pacs1* loss on synaptic transmission was evaluated in the L2/3 mPFC. Loss of *Pacs1* had no effect on mEPSC or mIPSC amplitude or frequency (Figs. S5f and g). Together, these findings suggest *Pacs1* loss does not significantly disturb excitatory or inhibitory synapses, supporting development of a PACS1 ASO strategy. They further suggest that PACS1 is an *in vivo* HDAC6 modulator, and that PACS2 compensates, to some extent, for loss of PACS1.

We found that a standard germline knock-in of R201W (equivalent to human R203W) caused embryonic lethality. Therefore, we adapted a modified gene trap strategy to conditionally express the knocked-in mutation from the endogenous *Pacs1* locus (Fig. S6a). We also generated Pacs1 WT mice harboring the gene trap while retaining Arg^201^ (Pacs1^M/+^), to control for haploinsufficiency. The gene-trapped lines were crossed with *Emx1*^*Cre*^ mice, permitting a direct comparison with the R26 lines, and which correctly removed the traps (Fig. S6a). Next, the effect of the R201W mutation on basal synaptic transmission was evaluated in L2/3 mPFC pyramidal neurons from the *Emx1*^*Cre*^-induced Pacs1^R201W/+^ and Pacs1^M/+^ mice. PACS1^R201W^ reduced mEPSC amplitude and frequency but had no effect on mIPSCs, similar to R26^P1R203W^ mice (Figs. 4a and S6b). Treatment of P1 pups with a Pacs1-specific ASO (P1ASO), which effectively and durably depletes PACS1 protein and mRNA (Figs. 4b and S6c), restored mEPSCs in juvenile PACS1^R201W/+^ mice to levels observed in the matched control Pacs1^M/+^ mice.

Recent studies suggest PACS1 forms a functional hub with PACS2 and WDR37, connecting the cellular chaperome to the membrane trafficking machinery ^11,23,24^. Indeed, recurrent missense mutations in each gene cause overlapping neurodevelopmental deficits ^5,25,26^. In support of this model, ASO depletion of Pacs1 profoundly reduced WDR37 protein levels (Fig. 4b). Similar results were observed in Pacs1^KO^ mice (Fig. S5e). Interestingly, PACS1 and WDR37 modulate calcium signaling by the ER, which is the cellular store that supplies calcium for action potential-independent mEPSCs ^21,27^. However, an aberrant PACS1^R203W^/WDR37 interaction alone is unlikely to be the sole underlying cause of PACS1 syndrome since the reduced mEPSC phenotype observed in R26^P1R203W^ mice was rescued by HDAC6 ASO, despite the presence of PACS1^R203W^ and WDR37 (Fig. 2e). Interestingly, PACS1^R201W^ suppressed PACS2 levels, which were restored by the P1ASO (Fig. 4b). Considering Pacs2 buffers the loss of Pacs1 (Table S2), these findings suggest Pacs1^R201W^ prevents induction of a compensatory role for PACS2. Strikingly, the Pacs1 ASO also reduced HDAC6 protein levels only in the Pacs1^R201W/+^ mice (Fig. 4b), suggesting the benefit achieved by the P1ASO on mEPSCs resulted, at least in part, from the coupled reduction of Pacs1^R201W^ and HDAC6.

Based on qPCR analysis (Fig. 4c), levels of PACS2, HDAC6 and WDR37 are post-transcriptionally controlled through pathways involving Pacs1- or Pacs1^R201W^. Interestingly, HDAC6 promotes liquid-liquid phase separation (LLPS) that underlies formation of membrane-less organelles, including stress granules (SGs) and aggresomes; SGs contain translationally stalled mRNAs, while aggresomes concentrate misfolded proteins for MT-based delivery to centrosome-associated bodies that are cleared by autophagy ^28–31^. Confocal analyses of patient cells revealed co-localization of HDAC6 with the SG marker, G3BP1, and in structures containing PACS1/PACS1^R203W^, which also contain p62 (Figs. 4d–f). Together, these findings suggest PACS1^R203W^ potentiates HDAC6 activity and induces LLPS in patient cells, potentially disturbing mRNA translation and clearance of aggregate-prone proteins.

Our studies suggest that a pathogenic interaction between PACS1^R203W^ and HDAC6 underpins PACS1 syndrome. The R203W change increases the multi-domain interaction between PACS1 and HDAC6, potentiating its deacetylase activity and pirating its posttranscriptional regulation. Together, PACS1^R203W^ and HDAC6 increase dendrite complexity and dendritic varicosities but reduce spine density and the number of functional synapses (see Fig. 4g). Speculatively, this PACS1^R203W^/HDAC6-induced dendritic overbranching, coupled with reduced spine density, suggests PACS1^R203W^ may induce early onset excitotoxic epileptic seizures that resolve postnatally when spine density is reduced ^32,33^. Indeed, PACS1 syndrome children frequently present with ID and early onset epilepsy that subsequently resolves, and both pathologies are associated with reduced spine density ^16,34^. The PACS1^R203W^-associated varicosities suggest a possible PACS1^R203W^/HDAC6-induced degenerative process that is frequently described in epilepsy, infantile neurobehavioral failure, and neurodegenerative disorders ^35–37^. The co-localization of PACS1^R203W^ with HDAC6 or p62 in aggresome-like structures supports this possibility. Finally, our study suggests PACS1 syndrome may be treatable with targeted therapies, including HDAC6 inhibitors or RNA-based strategies that deplete HDAC6 or PACS1 ^9,38^. We anticipate similar strategies may be applied to combat the many other recently identified NDDs also caused by recurrent missense mutations.

## Methods

### Experimental Animals:

All animal protocols were approved by the University of Pittsburgh Institutional Animal Care and Use Committee. Animals were housed in a temperature-controlled room and kept on a 12 h light/dark cycle with food and water available ad libitum. C57BL/6J mice (#:000664, RRID:IMSR_JAX:000664) and B6.129S2-*Emx1*^*tm1(cre)Krj*^/J mice (#005628, RRID:IMSR_JAX:005628) were from Jackson Labs. Pacs2^KO^ mice were described ^39^. All other engineered mice were generated by the Innovative Technologies Development and Mouse Embryo Services Cores in the Department of Immunology of the University of Pittsburgh, using CRISPR/Cas9 technology directly in C57BL/6J zygotes. All single guide RNAs were produced as described ^40^. R26^P1^ (B6;*Gt(ROSA)26Sor*^*tm1(CAG-LSL-Pacs1)Gath*^/J, ROSA26-LSL-PACS1-WT), and R26^P1R203W^ (B6;*Gt(ROSA)26Sor*^*tm1(CAG-LSL-Pacs1-R203W)Gath*^/J, ROSA26-LSL-PACS1-R203W) lines were generated as described ^12^; the HA-tagged cDNA for WT human PACS1 or PACS1^R203W^ were cloned into the pR26-GFP-Dest (a gift from Ralf Kuehn (Addgene plasmid # 74283)). Pronuclei of fertilized embryos (C57BL/6J, The Jackson Laboratory), produced by natural mating, were microinjected with a mixture of 0.3 μM EnGen Cas9 protein (ΝΕΒ M0646T), Rosa26-1 sgRNA (21 ng/μl ≈ 0.66 μM) and either the WT or the R203W targeting vector plasmid (10 ng/μl). The Pacs1^Δ4bp/+^ allele (Pacs1^em1Gath^, Pacs1-4bp-del, Pacs1-KO) was generated by injecting C57BL/6J zygotes with EnGen Cas9 protein and the guide Pacs1-e4-17Rev target sequence (5’-TACAAGAACCGAACTATCTTGGG-3’, chr19:5,160,008–5,160,030, mouse GRCm38/mm10 assembly), which resulted in a 4 bp deletion leading to a frameshift null allele. Pacs1^R201W/+^ (Pacs1^tm1Gath^, Pacs1-LSL-R201W) lines was generated using the Easi-CRISPR strategy ^41^. Briefly, the zygotes were injected with 0.33 μM EnGen Cas9 protein, 21.23 ng/μl (~0.66 μM) sgRNA (Pacs1-i3-18rev, 5’-TAGCTGGACCTGAACCACCAAGG-3’ ¸ chr19:5,160,141–5,160,163, mouse GRCm38/mm10 assembly ) and 10 ng/μl Pacs1-R201W-cKO Megamer (IDT). Pacs1^M/+^ (Pacs1^tm2Gath^, Pacs1-LSL-R201) was identified in the process; the Lox-Stop-Lox cassette was inserted in the intron, but R201 remained unchanged. The sequence of the Pacs1-R201W-cKO-ssODN was: 5’-ttctctgaccaatcactcttacaaccaagagacgctaaagccactaacactcagtcctctagctggacctgaaccaccaATAACTTCGTATAGCATACATTATACGAAGTTATTAGGGCGCAGTAGTCCAGGGTTTCCTTGATGATGTCATACTTATCCTGTCCCTTTTTTTTCCACAGCTCGCGGTTGAGGACAAACTCTTCGCGGTCTTTCCAGTcGATTACAAGGACGACGATGACAAGTAGATAACTTCGTATAGCATACATTATACGAAGTTATgaattcaggttctggttgctatcctggttaaccatttagtctgttttcctcttcagtaccctcatttccttaagcgagatgccaacaaactgcagattatgttgcaaaggaggaagcggtacaagaacTgGacCatcttgggctataaaaccttagctgtgggactcatcaacatggcagaggtgagcagaacacaagtcttagactgcaggtct-3’. Oligonucleotides used for genotyping are presented in Table S3.

### ASOs and intracerebroventricular (ICV) injections:

The MOE [2Ȳ-O-(2-Methoxyethyl)] gapmer ASOs used in this study were generated as described ^42^. The negative ASO (nASO, 5Ȳ-CCTATAGGACTATCCAGGAA–3) and mouse HDAC6 ASO (H6ASO, 5’-GCCTACTCTTTCGCTGTC-3Ȳ) were previously reported ^42^, and the mouse PACS1 ASO (P1ASO, 5’-TCTCATTTTGACTATACCAT-3’) was screened and validated as described ^43^. ICV injections were performed on genotyped P1 pups as described ^44^. Pups were cryo-anesthetized for 8 min, and the injection site was located ~0.25 mm lateral to the sagittal suture and 0.5–0.75 mm rostral to the neonatal coronary suture. ASOs diluted to 20 μg/μl in PBS (no calcium or magnesium) containing 0.01% Fast Green were drawn into a Hamilton syringe with a 32g needle, positioned perpendicular to the skull surface, and impaled to a depth of 2 mm into the right hemisphere to deliver a single dose of 40 μg (2 μl injection) of ASO into the ventricle. After 10 seconds, the needle was slowly retracted to prevent backflow. The pups were gently warmed on a heating pad until fully recovered, returned to the nest, and monitored daily.

### Slice electrophysiology:

Animals were anesthetized with isoflurane and then decapitated. Brains were quickly removed and placed in ice-cold NMDG-HEPES aCSF pH 7.3 (92 mM NMDG, 2.5 mM KCl, 1.25 mM NaH2PO4, 30 mM NaHCO3, 20 mM HEPES, 25 mM glucose, 2 mM thiourea, 5 mM Na-ascorbate, 3 mM Na-pyruvate, 0.5 mM CaCl2, and 10 mM MgSO4, pH adjusted to 7.3 with HCl, osmolarity 305 mOsm, and saturated with 95% O2 /5% CO2). Coronal slices (250 μm) were cut with a vibratome (Leica VT1200s) and allowed to recover first in oxygenated NMDG-HEPES at 37 °C for 9 minutes, then transferred to HEPES-aCSF (86 mM NaCl, 2.5 mM KCl, 1.2 mM NaH2PO4, 35 mM NaHCO3, 20 mM HEPES, 2 mM CaCl2, 1 mM MgSO4, 25 mM glucose, 5 mM sodium ascorbate, 2 mM thiourea, and 3 mM sodium pyruvate, pH adjusted to 7.3, osmolarity 305 mOsm) at room temperature for at least one hr. Recovered slices were transferred to a recording chamber mounted on an Olympus BX61WI microscope, under continuous perfusion (2 ml/min) with oxygenated artificial CSF (aCSF: 119 mM NaCl, 2.5 mM KCl, 1 mM NaH2PO4, 1.3 mM MgCl2, 2.5 mM CaCl2, 26.2 mM NaHCO3, and 11 mM glucose, osmolarity 290 mOsm, saturated with 95% O2/5% CO2) heated to 29 ± 2°C via an in-line heater (Warner Instruments). Whole-cell recordings were made from visually identified mPFC L2/3 pyramidal neurons. The extracellular solution contained TTX (1 μM, tetrodotoxin citrate, Hello Bio) and D-AP5 (100 μM, Hello Bio) to block action potentials and NMDA receptor activities respectively. The intracellular solution contained 108 mM Cs-gluconate, 20 mM HEPES, 5 mM TEA-chloride, 2.8 mM NaCl, 0.4 mM EGTA, 5 mM BAPTA, 4 mM MgATP, 0.3 mM NaGTP, pH 7.2 and 290 mOs. mEPSCs were recorded as inward currents at a holding potential of −72 mV, and mIPSCs were recorded as outward currents at a holding potential of 0 mV. After acquisition of a stable baseline (~8 min), miniature postsynaptic events were recorded for ~8 min at each potential. Recordings were excluded if the access resistance was > 25 MΩ or changed more than 20% during the recordings. All signals were filtered at 2.6 KHz, amplified at 5x using a MultiClamp 700B amplifier (Molecular Devices), and digitized at 20 KHz with a Digidata 1440A analog-to-digital converter (Molecular Devices).

### Immunohistochemistry:

Mice were anesthetized with ketamine (90 mg/kg)/ xylazine (20 mg/kg) and transcardially perfused with ice-cold 4% paraformaldehyde (PFA). Brains were removed, post-fixed overnight in 4% PFA at 4° C, dehydrated in 30% sucrose and embedded in OCT (Tissue Tek). 35 μm sections were prepared on a Leica CM1950 cryostat. Staining was performed on free-floating sections without antigen retrieval. Sections were washed 3x with 0.1% Triton-X100 in PBS and then blocked for 1 h with 5% normal goat serum (NGS), 1% bovine serum albumin (BSA) and 0.3% Triton-X100 in PBS at RT. Primary antibodies were diluted in 0.1% Triton-X100 and 5% NGS, and sections were incubated overnight at 4°C under gentle agitation. Sections were then washed 3x (10 min/wash) with 0.3% Triton-X100 and 1% NGS in PBS, and incubated with species-specific secondary antibodies diluted in PBS containing 1% bovine serum albumin (BSA), 2% NGS and 0.1% Triton-X100 for 2h at RT. Nuclei were counterstained with Hoechst 33342. Sections were mounted onto glass slides using Fluoromount-G (Invitrogen, 00-4958-02), cured and imaged.

### Dendrite arbor reconstructions and spine density analysis.

P1 pups were ICV-injected with 3×10^8^ Paav-FLEX-tdTomato viral particles (Addgene #28306-AAV9) combined with 40 μg of ASOs, as detailed above. At P18, mice were anesthetized with ketamine/xylazine and transcardially perfused with ice-cold 4% paraformaldehyde (PFA). Brains were removed and post-fixed overnight in 4% PFA at 4° C under gentle agitation. For dendritic reconstructions, 250 μm coronal slices were sectioned using a Leica VT1000S vibratome. Slices containing the hippocampus were incubated with a 1:800 Hoechst 33342 solution in PBS for 10 min at RT under gentle agitation and then washed 3X with PBS. Slices were subsequently mounted onto glass slides with Fluoromount-G. CA1 pyramidal tdTomato^+^ neurons were identified based upon the cell body location relative to other hippocampal areas. Full z-stack images were acquired on a Nikon A1R confocal Ti2-E microscope. Dendritic arbors were traced using the Simple Neurite Tracer (SNT) plugin of the Fiji software, as previously described ^45^. Sholl analysis, total dendrite length, and branching point number were subsequently quantified using the same software. For spine density analysis, 50 μm coronal slices were acquired on a vibratome. Sections containing the hippocampus were blocked for 1 h in 5% normal goat serum (NGS), 1% bovine serum albumin (BSA) 0.3% Triton-X100 in PBS at RT. Sections were then incubated overnight at 4°C with rabbit anti-RFP. Sections were washed three times for 10 min and incubated for 90 min with goat anti-rabbit Alexa Fluor 488. Brain sections were washed 3X and counterstained with Hoechst 33342. Spines on secondary apical dendrites of CA1 hippocampal pyramidal neurons were imaged and analyzed with NeuroLucida360 and the dendritic length was calculated using NeuroExplorer 360 as described ^46^.

### Neuron Isolation and Culturing:

Primary mouse hippocampal neurons were prepared from P1 *Emx1*^*Cre/+*^*; R26*^*P1*^ or *Emx1*^*Cre/+*^*; R26*^*P1R203W*^ mice as described ^47^ with a few modifications. Pups were anesthetized in ice for 9 min, brains were harvested, and hippocampi were micro-dissected. Excised hippocampi were washed and resuspended in ice-cold HBSS (Gibco, 14175–095) supplemented with 1 mM sodium pyruvate (Gibco, BW13115E), 0.1% w/v glucose (Sigma, G6152) and 10 mM HEPES pH 7.3 (Gibco, 15630–080), followed by a 15 min digestion by papain (Worthington, LK003178) and 6 μg/ml DNase I (Sigma, DN25) supplemented with 2 mM MgSO4 (Sigma, M2643) at 37 °C with gentle agitation. Dissociated cells were pelleted and resuspended in NeuroBasal medium (Gibco, 21103049), gently triturated using polished Pasteur pipettes and filtered through a 70 μm nylon strainer. Cells were counted using Trypan Blue, washed, and resuspended in NeuroBasal maintenance medium supplemented with B-27 (Gibco, 17504044), GlutaMax (Gibco, 35050061) and penicillin/streptomycin (Lonza, 17-602E). 4 × 10^4^ cells were seeded onto 18 mm coverslips washed with 70% nitric acid (J.T. Baker, #959800) and thinly coated with Matrigel (Corning, 354277) in a 12-well plate. Six hr after plating, half of the medium from each well was carefully removed and replaced with fresh maintenance medium. At DIV2 (two days post-plating), cells were treated with 2 mM AraC (Sigma, C6645) to reduce glial cell growth. Maintenance medium was replenished every 3–4 days until cells were processed for imaging.

### Confocal Microscopy:

Images were captured using a Nikon Ti2-E confocal microscope with a resonant scanner and processed using the Nikon Elements analytical package. Analysis of Golgi localization and fragmentation was designed and automated using Nikon Elements software. In fibroblasts, Giantin (Golgi marker) staining that extended > 10 μm from the nuclear margin was considered peripheral Golgi. In primary neurons, deployment of the Golgi apparatus was determined by measuring the distance from the nuclear margin to the most distal end of the Giantin-positive emanating Golgi process.

### qPCR:

RNA was isolated from micro-dissected mouse cortex using an RNeasy Mini Kit (QIAGEN, 74104). cDNA was prepared using SuperScript IV First-Strand Synthesis System Kit (Invitrogen, 18090050). qPCR reactions were performed in a QuantStudio 3 Real-Time PCR System (Applied Biosystems, A28567) with the PowerUp SYBR Green PCR Master Mix (Applied Biosystems, A25776) and the primer pairs listed in Table S3. All reactions were performed in triplicate. qPCR primers used in this study are listed in Table S4.

### Brain harvest and lysate preparation.

Animals were anesthetized with isoflurane and then decapitated. Brains were removed, immediately frozen in liquid nitrogen, and stored at −80C until further processing. Cortices were homogenized in ice-cold RIPA buffer (1% Triton-X100, 0.1% SDS, 1% DOC, 50 mM Tris-HCl pH 7.4, 150 mM NaCl and 1 mM EDTA) containing freshly added protease/phosphatase inhibitors. For acetylated protein blotting, 5 μM trichostatin A (TSA) was added into the lysis buffer. Protein concentration of the brain lysates was determined by Bradford method according to manufacturer’s instructions (Bio-Rad). For Western blots, 15–30 μg of protein was separated on 6–12% SDS-PAGE gels.

### Synaptosome Fractionation:

Synaptosome fractions were prepared essentially as described ^48^. Whole brains were harvested from C57BL/6 mice and homogenized in 50 mM Tris-acetate pH 7.4, 10% w/v sucrose, and 5 mM EDTA containing protease inhibitors (0.5 mM PMSF, 0.1 mM Aprotinin, 0.25 mM Pefabloc, 3 μM E-64 and 0.1 mM Leupeptin) and phosphatase inhibitors (1 mM Na_3_VO_4_ and 20 mM NaF) using a Teflon glass homogenizer. Lysates were centrifuged at 800 x g for 20 min, and the supernatant (cytosol fraction) was transferred to a new tube and sedimented at 16,000 x g for 30 min. The supernatant was saved as (non-synaptosomal fraction), and the pellet was resuspended in hypotonic buffer (5 mM Tris-Acetate, pH 8.1), incubated on ice for 45 min, homogenized in a Teflon glass homogenizer and mixed with 51% w/v sucrose to a final concentration of 34% w/v sucrose. An equal volume of 28.5% and 10% w/v sucrose cushion in 50 mM Tris-acetate (pH 7.4) was sequentially layered onto the extract, and the 3-layer gradient was sedimented at 60,000 x g for 2 h. The protein-enriched layer was collected at the interface between the 34% and 28.5% w/v sucrose layers, diluted with 50 mM Tris-Acetate pH 7.4 to 10% w/v sucrose. The sample was sedimented at 48,000 x g for 30 min, and the pellet was resuspended in 50 mM Tris-acetate pH 7.4 plus 0.1% SDS (synaptosomal fraction). Protein concentration was determined using the Bradford method according to manufacturer’s instructions (Bio-Rad, 500–0205). Four micrograms of total protein from each fraction were separated on 10% SDS-PAGE gels for Western blot analyses.

### Immunoprecipitation and Western blot:

For most immunoprecipitation experiments, HCT116 cells were transfected with plasmids using lipofectamine 2000. After 24 hr, cells were lysed in harvest buffer (50 mM Tris-Cl pH 7.4, 150 mM NaCl, 1% NP-40 and 10% glycerol) containing protease inhibitors (0.5 mM PMSF, 0.25 mM Pefabloc, 0.1 mM Aprotinin, 3 μM E-64 and 0.1 mM Leupeptin) and phosphatase inhibitors (1 mM Na3VO4 and 20 mM NaF). Lysates were precleared at 16,000 x g for 15 min, and 10% of supernatant was mixed with 5x Laemmli sample buffer as input control, while the remaining lysate was incubated with a 50% slurry anti-flag M2 affinity beads (Sigma, A2220) at 4 °C overnight. The beads were washed three times with modified RIPA buffer (50 mM Tris-Cl pH 8.0, 150 mM NaCl, 1% NP-40 and 1% DOC) and captured proteins were eluted in 2x Laemmli sample buffer at 95 °C for 5 min. The input control and the IP eluate were separated on SDS-PAGE gels for Western blot analyses. Western blots were developed with the Pierce ECL Western Blotting Substrate (Thermo Fisher) using a FluorChem E image acquisition system (ProteinSimple). Signals were quantified using the AlphaView image analysis software package (ProteinSimple).

### Cell Lines, Antibodies and Chemicals:

Cell lines- PACS1 syndrome dermal fibroblasts GM27159 (R203W patient), GM27160 (parent), GM27650 (R203W patient) and GM27651 (parent) were from Coriell Institute and immortalized by transduction with hTERT (LVP1130-Puro, Gentarget). Pacs1^WT^ and Pacs1^Δ4bp/Δ4bp^ embryonic fibroblasts were isolated from E13.5 littermate embryos and immortalized with a retrovirus expressing SV40 large T antigen (kindly provided by M. Suda, UPMC). HCT116 cells were maintained as described ^39^. All cell lines were passaged in DMEM + 10%FBS and pen/strep. Antibodies-actin (Millipore, MAB1501), α-actinin (Cell Signaling Technology (CST) 3134S) α-tubulin (DMA1 Cell Signaling 3873S and Thermo Fisher 66031), Ac-Lys^40^-α -tubulin (CST 5335S), cortactin (4F11, Sigma 05–180), Ac-cortactin (Sigma 09–881), CTIP2 (25B6 Abcam 18465), SATB2 (Abacm 51502), EB1 (BD Transduction 610534), G3BP1 (CST 17798), V5 (Invitrogen, R960-25), HDAC6 (Abcam 253033 and D2E5 CST 7558S), p62 (Abcam 56416), Flag (Sigma-Aldrich, F7425 and A2220), HA (CST 3724S and Biolegend 901513), furin (MON-152, kindly provided by J. Creemers, Leuven), GAPDH (14C10 CST 2118S), Giantin (kindly provided by Dr. A. Linstedt, CMU), pericentrin (AbCam 4888), MAP2 (Biolegend 801810), βIII-tubulin (Biolegend 801213), PSD95 (NeuroMab 75-028-020), GABA_A_Rα1 (NeuroMab 75-136-020), AMPAR1 (CST 13185), HDAC6 (CST 7558S, Assay BioTech C0226, and Abcam 253033), WDR37 (Sigma HPA037565), RFP (Rockland 600-401-379), PACS1 (BD Transduction Labs 611371, Invitrogen PA558589, and ^49^), PACS2 ^39^, Goat anti-Rabbit IgG Alexa Fluor 488 (Invitrogen A11008), Goat anti-Mouse IgG1 Alexa Fluor 568 (Invitrogen A11004) Goat anti-Mouse IgG Alexa Fluor 647 (Invitrogen A-21242), Goat anti-Chicken IgY Alexa Fluor 633 (Invitrogen A-21103), Goat anti-Rat IgG Alexa Fluor 488 (Invitrogen A-11006), Goat anti-Rabbit IgG Alexa Fluor 594 (Invitrogen A-11012). Chemicals- Nocodazole (Sigma 487929), Tubacin (Cayman Chemical NCO778559), SW-100 (MCE HY-115475), ACY-1215 (MCE HY-16026), AGK2 (Sigma A8251), TTX (Hello Bio HB1035), D-AP5 (Hello Bio HB0225; Tocris Cat. No. 0106).

### Plasmids, siRNAs, and AAV:

**Figure 1:**
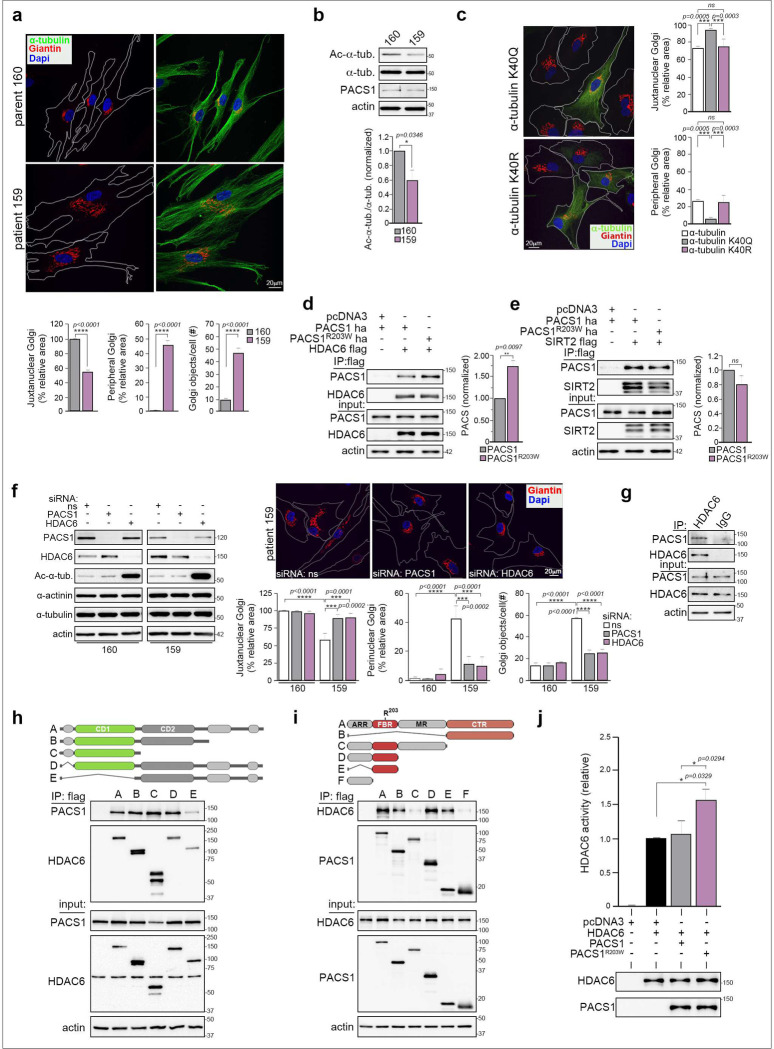
PACS1^R203W^ interacts with HDAC6 to increase enzyme activity and disturb Golgi positioning. **(a)** (Top) PACS1^R203W^ patient (159) and healthy parent (160) fibroblasts were fixed and stained for the Golgi-marker Giantin (red), α-tubulin (green) and nuclei (DAPI). (Bottom) Quantification of Golgi fragmentation (number of Golgi objects per cell) and dispersal, expressed as percentage of total Golgi area within 10 μm of nuclear envelope (Juxtanuclear) or not (Peripheral). Data are mean ± SEM, n = 36–37 cells/group. **(b)** Western blot of total α-tubulin and Ac-Lys^40^-α-tubulin in 159 and 160 fibroblasts. Data are expressed as normalized mean ± SD, n=3. **(c)** Patient (159) cells expressing GFP-tagged K^40^Q-α-tubulin or K^40^R-α-tubulin were fixed and stained for Giantin (red), GFP (green) and nuclei (DAPI). Golgi area in GFP-positive or adjacent control cells (white bars) was measured as in (a). Data are mean 00B1 SD, n=3. **(d and e)** FLAG-tagged HDAC6 (d) or FLAG-tagged SIRT2 (e) were co-transfected with HA-tagged PACS1 or PACS1^R203W^ as indicated. FLAG-tagged proteins were immunoprecipitated and co-precipitating PACS1 proteins were detected by Western blot (anti-HA). **(f)** 159 and 160 cells, nucleofected with a non-specific siRNA (NS) or PACS1- or HDAC6-targeting siRNA, were harvested for Western blot (left) or processed for confocal imaging (right) and analyzed as in (a), after staining for Giantin (red) and nuclei (DAPI). Data are mean 00B1 SD, n=3. See also Fig. S2b. **(g)** Endogenous PACS1 was immunoprecipitated from HCT116 cells and co-precipitating HDAC6 was detected by Western blot. IgG was used as a negative control. **(h-i)** HCT116 cells co-expressing (h) PACS1-HA and the indicated FLAG-tagged HDAC6 constructs or (I) HDAC6-V5 and the indicated FLAG-tagged PACS1 constructs were harvested, and FLAG-tagged proteins were captured with M2 agarose. Co-precipitating PACS1-HA (H) or HDAC6-V5 (i) was detected by Western blot. PACS1 regions; ARR, atrophin-related region; FBR, furin(cargo) binding region; MR middle region; CTR, C-terminal region. **(j)** Cells expressing HDAC6-FLAG alone or together with HA-tagged PACS1 or PACS1^R203W^ were lysed, and HDAC6-FLAG was immunoprecipitated with M2 agarose. Bound proteins were eluted with FLAG peptide, and equal amounts of HDAC6 from each sample .were used to measure deacetylase activity using the Fluor-de-Lys substrate. Data are mean ± SD, n=3.

Plasmids- pHDAC6 flag was kindly provided by J. Hu (University of Pittsburgh), pEGFP Tubulin K^40^Q and K^40^R were gifts from Kenneth Yamada (Addgene plasmids #105302 and #105303; RRIDs:Addgene_105302 and Addgene_105303), pSIRT2 Flag was a gift from Eric Verdin (Addgene plasmid # 13813; RRID:Addgene_13813), pPACS-1-FLAG and pPACS-1-HA have been described ^49^. siRNAs- Human PACS1 siRNA (L-006697-01), human HDAC6 siRNA (L-003499-00) and the non-targeting control RNA (D-001810-10, (Horizon/Dharmacon ON-TARGETplus) were nucleofected (Amaxa) into cells as described ^39^. AAV- pAAV-FLEX-tdTomato was a gift from Edward Boyden (Addgene viral prep # 28306-AAV9; RRID: Addgene 28306). Flag-tagged PACS1^R203W^ was made by site-directed mutagenesis using standard PCR methods. Other plasmids were generated by Gibson Assembly (GA) cloning using GA reaction mix (NEB M5510AA) and gBlocks (IDT). These include HDAC6-V5 as well as FLAG-tagged HDAC6^1–465^, HDAC6^1–864^, HDAC6^Δ4–85^, HDAC6^Δ4–476^ (see Fig. 1 panel I, constructs B-E, respectively), and also Flag-tagged PACS1^Δ5–542^, PACS1^1–542^, PACS1^1–266^, PACS1 ^Δ5–117^, PACS1^1–117^ and PACS1^1–266R203W^ (see Fig. 1 panel j constructs B-F and Fig. S2c, respectively).

### HDAC6 activity:

HDAC6 activity was measured essentially as described ^50^. Briefly, HDAC6/f was immunoisolated from transfected cell lysates using anti-FLAG M2-affinity gel (Millipore A2220). The beads were washed and bound HDAC6/f was eluted with FLAG peptide (Millipore F3290). Following normalization of immunoreactive protein by Western blot, HDAC6/f-containing eluants were incubated with 50 μM FLUOR-DE-LYS peptidyl substrate (Enzo BMLKI1040050) for 1 hr at 37°C in activity buffer (140 mM HEPES, 10 mM KCl, 1 mg/ml BSA 1 mM βME, pH 7.4). Fluorescent AMC was released from the deacetylated substrate by digesting with Trypsin-TPCK (Sigma T1426) in resolving buffer (20 mM Tris, 150 mM NaCl, 1 mM EDTA, pH 7.4). AMC signals were detected using a Synergy 4 microplate reader (λ_ex_ = 420 nm and (λ_em_ = 460 nm). Controls included no HDAC6/f or addition of 5 μM tubacin.

## Supplementary Material

Supplement 1

## Figures and Tables

**Figure 2:**
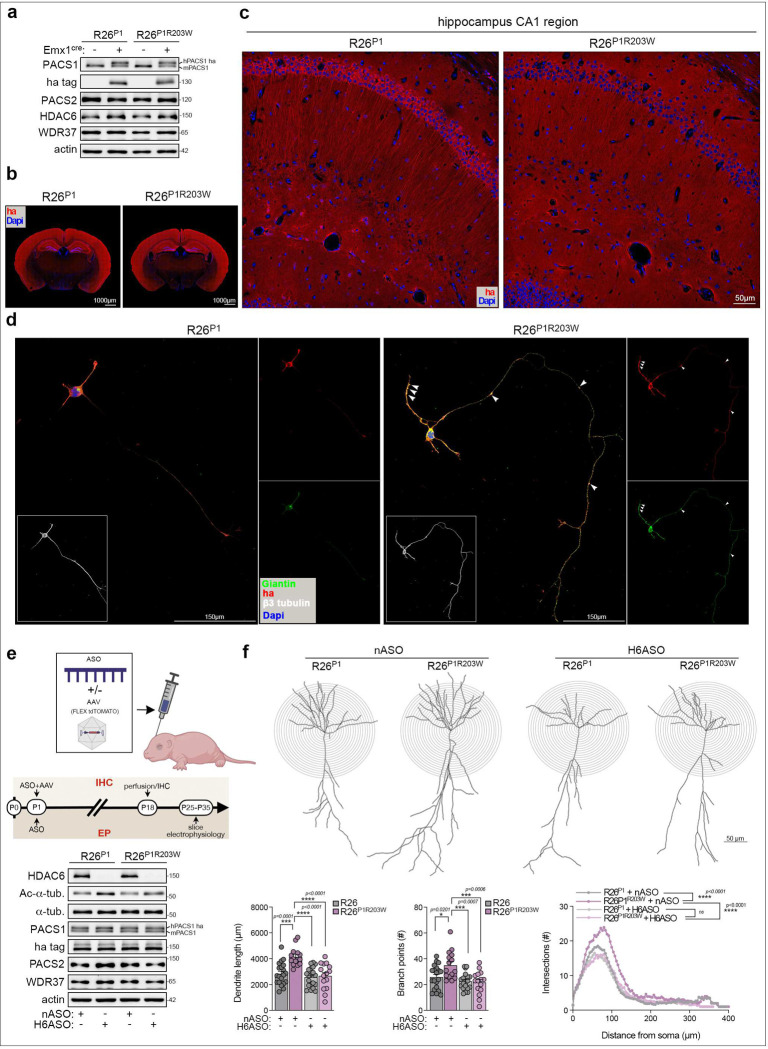
HDAC6 ASO reverses the PACS1^R203W^/HDAC6-dependent increased dendrite complexity in hippocampus. (a and b) Western blots (a) and immunohistochemistry (IHC) of coronal brain sections (b) prepared from adult R26^P1^ and R26^P1R203W^ lines crossed or not with *Emx1*^*Cre*^ mice to induce expression of HA-tagged PACS1 or PACS1^R203W^ (red). Scale bar, 1,000 μm. **(c)** Hippocampal CA1 region from specimens in (b) demonstrating the differential subcellular localization of HA-tagged PACS1 and PACS1^R203W^ (red). Scale bar, 50 μm. **(d)** Dissociated hippocampal neurons, isolated from *Emx1*^*Cre*^*;R26*^*P*1^ or *Emx1*^*Cre*^*;R26*^*P1R203W*^ mice at P0, were processed for confocal imaging at DIV 5 to detect β3-tubulin (magenta, pseudocolored white), Giantin (green) and HA-tagged PACS1 or PACS1^R203W^ (red). Arrow heads indicate colocalization of Giantin and PACS1R203W in varicosities along the developing neurites. Scale bar, 150 μm. **(e and f)** P1 *Emx1*^*Cre*^*;R26*^*P*1^ and *Emx1*^*Cre*^*;R26*^*P1R203W*^ pups were injected ICV with nASO or H6ASO (40 μg) together or not with Cre-inducible AAV9-FLEX-tdTomato (1×10^8^ vp). As depicted in the timeline (e), isolated cortices were processed for IHC and Sholl analysis of tdTomato-filled CA1 hippocampal pyramidal neurons (IHC; see panel f), or electrophysiology (EP; see Fig. 3), and Western blot (e). Data are mean ± SEM, n = 15–20 neurons/group, and 3–5 animals per condition (2-way ANOVA).

**Figure 3:**
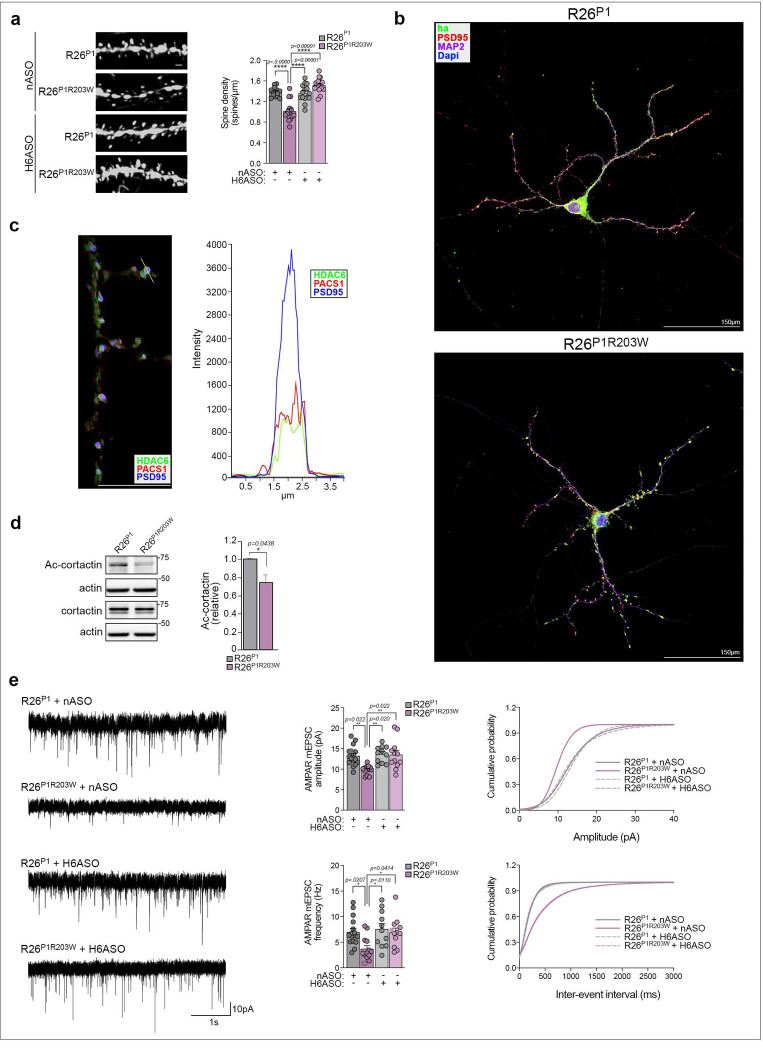
HDAC6 ASO rescues PACS1^R203W^/HDAC6-induced deficits in spine density and spontaneous release. **(a)** (Left) Representative images of secondary dendrites from the specimens in Fig. 2F with a spine density < 1 SD away from the group mean. Scale bar 1 μm. (Right) Data are average group value ± SEM (2-way ANOVA) for 4–6 dendrites per neuron, 4–6 neurons per animal, and 3 animals per condition. **(b)** Dissociated hippocampal neurons isolated from P0 *Emx1*^*Cre*^:R26^P1^ and *Emx1*^*Cre*^:R26^P1R203W^ mice were processed for confocal imaging at DIV18 to detect MAP2 (magenta), HA-tagged PACS1 or PACS1^R203W^ (green) and PSD95 (red). Scale bar 150 μm. **(c)** DIV18 neurons prepared from WT C57BL/6 mice were processed for confocal imaging to detect endogenous HDAC6 (green), PACS1 (red) and PSD95 (blue), and fluorescent signal from the indicated spine (white line) was measured (Nikon Elements). Scale bar 10 μm. **(d)** Western blot of Ac-cortactin, total cortactin and actin in cortex isolated from R26^P1^ or R26^P1R203W^ mice. Data are mean ± SD, n=3. **(e)** (Left) Representative whole-cell voltage-clamp recordings of mEPSCs from L2/3 pyramidal neurons in acute brain slices of juvenile *Emx1*^Cre^-induced R26^P1^ and R26^P1R203W^ mice injected (ICV) at P1 with 40 μg nASO or H6ASO (see Fig. 2e timeline). (Middle) Summary data of AMPAR mEPSC amplitude (top) and frequency (bottom). Data are mean ± SEM. (Right) Cumulative probability distributions of AMPAR mEPSC amplitudes (top) and frequencies (bottom). n = 11–16 neurons/group, and 3–5 animals per condition (2-way ANOVA).

**Figure 4:**
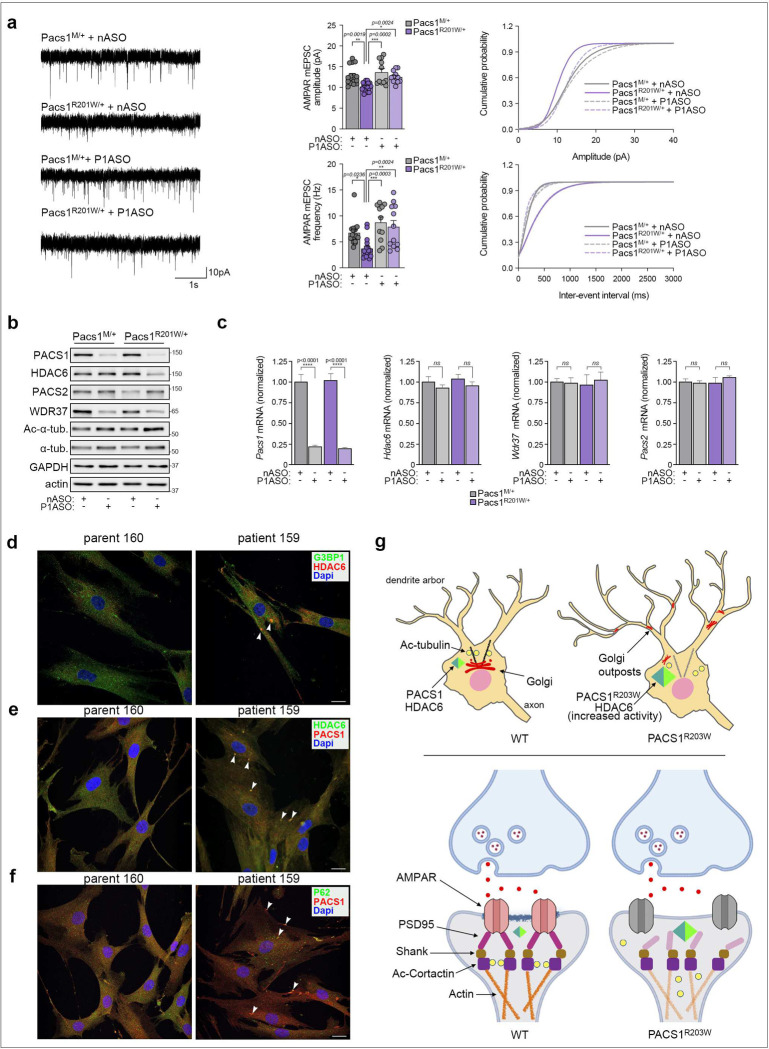
PACS1 ASO rescues PACS1^R201W^-induced deficits in spontaneous release but disturbs posttranscriptional gene regulation. **(a)** (Left) Representative whole-cell voltage-clamp recordings of mEPSCs from L2/3 pyramidal neurons in acute brain slices of juvenile *Emx1*^*Cre*^-induced Pacs1^R201W/+^ and Pacs1^M/+^ control mice injected (ICV) at P1 with 40 μg nASO or P1ASO. (Middle) AMPAR mEPSC amplitude (top) and frequency (bottom) of *Emx1*^*Cre*^-induced Pacs1^R201W/+^ or Pacs1^M/+^ mice injected at P1 with nASO or P1ASO. Data are mean ± SEM. (Right) Cumulative probability distributions of AMPAR mEPSC amplitudes (top) and frequencies (bottom). n = 11–17 neurons/group, and 4–6 animals per condition (2-way ANOVA). **(b)** Western blot of cortical lysates from mice in panel A. **(c)** qRT-PCR of cortex RNA encoding PACS1, PACS2, HDAC6 and WDR37 isolated from the ASO-treated *Emx1*^*Cre*^*;Pacs1*^*M/+*^ and *Emx1*^*Cre*^*;Pacs1*^*R201W/+*^ mice. Data are mean ± SEM, n= 4 mice per group. **(d-f)** PACS1^R203W^ patient (159) and healthy parent (160) fibroblasts were fixed and stained for (**d**) HDAC6 (red), G3BP1 (green) and nuclei (DAPI), **(e)** PACS1 (red), HDAC6 (green) and nuclei (DAPI), or **(f)** PACS1 (red), p62 (green) and nuclei (DAPI). Arrowheads indicate co-localization. **(g)** Working model for the effect of PACS1^R203W^ on dendritic arborization and spine organization. (Top) In healthy neurons, acetylated MTs stabilize the Golgi ribbon in the cell body. In PACS1 syndrome neurons, PACS1^R203W^/HDAC6 causes excessive deacetylation of MTs, leading to Golgi fragmentation and the redistribution of Golgi outposts into dendrites, which undergo overbranching. (Bottom) In healthy neurons, Ac-cortactin stabilizes the PSD95-containing subsynaptic scaffold that organizes functional cell-surface AMPA receptors. In PACS1 syndrome neurons, PACS1^R203W^/HDAC6 causes excessive deacetylation of cortactin, disorganizing the postsynaptic scaffold and reducing AMPA receptor-mediated mEPSCs. It is not known if PACS1^R203W^/HDAC6 causes lateral diffusion or internalization of the affected AMPARs. Created with Biorender.com.

## Data Availability

Patient cell lines are available from Coriell Institute. All plasmids, ASOs and mouse lines developed during this study will be openly shared with proper material transfer agreements in place with the University of Pittsburgh or IONIS Pharmaceuticals.
