# Supplementary material for: RNA-targeted therapy corrects neuronal deficits in PACS1 syndrome mice
Source: Res Sq. 2023 Jan 27:rs.3.rs-2440581. Preprint. [Version 1] doi: 10.21203/rs.3.rs-2440581/v1 (PMC9901029; doi:10.21203/rs.3.rs-2440581/v1)
Supplement: Supplement 1 [file NIHPPRS2440581v1-supplement-1.pdf]

Table S1

Genotype of pups at weaning from *Pacs1*^{HET} x *Pacs1*^{HET} breeding

	Genotype			
	Pacs1^{WT}	*Pacs1*^{HET}	*Pacs1*^{KO}	Total
E	74.75	149.5	74.75	299
O	78	168	53	299

E: Expected; *O*: Observed. χ^2 = 8.76 p = 0.013

Table S2

Genotype of pups at weaning from *Pacs1*^{HET}; *Pacs2*^{HET} x *Pacs1*^{HET}; *Pacs2*^{HET} breeding

N = 121			*Pacs1* alleles		
			Pacs1^{WT}	*Pacs1*^{HET}	*Pacs1*^{KO}
Pacs2 alleles	*Pacs2*^{WT}	*E*	7.56	15.13	7.56
		O	13	18	7
	Pacs2^{HET}	*E*	15.13	30.25	15.13
		O	21	38	7
	Pacs2^{KO}	*E*	7.56	15.13	7.56
		O	5	12	0

E: Expected; *O*: Observed. $\chi^2 = 22.21$, $p = 0.0045$

Table S3

Genotyping PCR primers	
**Primer Name**	**Sequence (5' - 3')**
e4F	GGTACAAGAACCGAACTATCT
eD4F	GGTACAAGAACCGAACTTGG
i4R	CTGGTCTACAAAGTGAGTTCC
NeoF	GCTAACCATGTTCATGCCTTC
NeoR	CGTTGGCTACCCGTGATATT
R26F	GCCTCCTGGCTTCTGAGGACCG
R26R	TCTGTGGGAAGTCTTGTCCCTCC
SAR	CCTGGACTACTGCGCCCTACAGA
GFPF	AGGACGACGGCAACTACAAG
GFPR	GTCCATGCCGAGAGTGATCC
i3F	TCGTCATAATCCTGGTGACTGT
e4R	CCTGCAGGCTGGAGAAACA

Table S4

RT-qPCR primers	
**primer name**	**sequence (5' - 3')**
Gapdh F	CATGGCCTTCCGTGTTCCTA
Gapdh R	GCCTGCTTCACCACCTTCTT
Pacs1 F	AGACACCACCAGTCCTATGG
Pacs1 R	GCAGATCAGCTTTGCTGGAC
Hdac6 F	CACCGCATTCAGAGGGTTCT
Hdac6 R	CCTTAAGGTGGGGCCAGAAG
Pacs2 F	GAAAACCCAAGAAGCAGCGG
Pacs2 R	GCTCCGAATCCAAGACCTCC
Wdr37 F	ACCACATCCAGGGCCATCTGT
Wdr37 R	CTATGCTCCACAACAAAGCCGT

Supplemental Figure Legends

Figure S1: (a) (Top) PACS1^{R203W} patient (650) and healthy parent (651) fibroblasts were fixed and stained for Giantin (red), α-tubulin (green) and nuclei (DAPI). (Bottom) Quantification of Golgi fragmentation and dispersal, as described in Fig. 1. Data are mean ± SEM, n = 30-50 cells/group. **(b)** Patient (159) and parent (160) cells were exposed to 5 µM nocodazole for 10 hr to depolymerize MTs. After a 3-min washout, cells were fixed and stained for EB1 (green), Pericentrin (red), and nuclei (DAPI). Arrows indicate asters. Data are mean ± SD, n = 50 cells /group. **(c)** Western blot of total α-tubulin and Ac-Lys^{40}-α-tubulin in 651 and 650 fibroblasts. Data are mean ± SD, n=3.

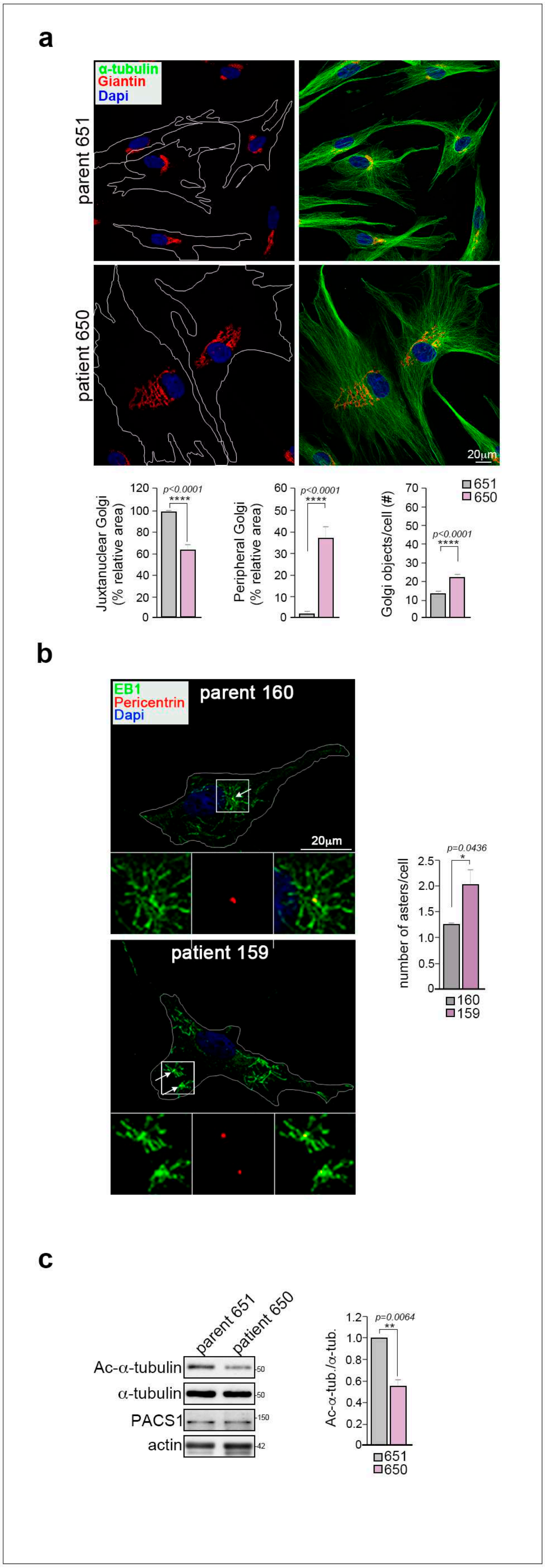
a
α-tubulin
Giantin
Dapi
parent 651
patient 650
20μm
Juxtanuclear Golgi (% relative area)
p<0.0001
****
Peripheral Golgi (% relative area)
Golgi objects/cell (#)
651
650
b
EB1
Pericentrin
Dapi
parent 160
patient 159
20μm
number of asters/cell
p=0.0436
*
160
159
c
parent 651
patient 650
Ac-α-tubulin
α-tubulin
PACS1
actin
Ac-α-tub./α-tub.
p=0.0064
**

Fig S1

Figure S2: (a) (Top) Patient (159) and parent (160) cells were treated with vehicle alone (DMSO) or with HDAC6 inhibitors, including tubacin (5 µM, 4 h), SW-100 (5 µM, 16h), or ACY1215 (2.5 µM, 16 h), or the SIRT2 inhibitor AGK2 (10 µM, 16 h). Cells were fixed and stained for Giantin (red) and nuclei (DAPI). (Bottom) Quantification of Golgi fragmentation and dispersal, as described in Fig. 1. Data are mean ± SEM, n= 30-100 cells for each condition from 3 independent experiments. **(b)** Confocal images of 160 cells nucleofected with the NS, PACS1 or HDAC6 siRNAs described in Figure 1F. **(c)** HCT116 cells co-expressing HDAC6-V5 and the FLAG-tagged PACS1$^{1\text{-}266}$ (construct D in Figure 1I) or R203W-PACS1$^{1\text{-}266}$ constructs were harvested and FLAG-tagged proteins captured with M2 agarose. Co-precipitating HDAC6-V5 was detected by Western blot. Data are mean ± SD, n = 3.

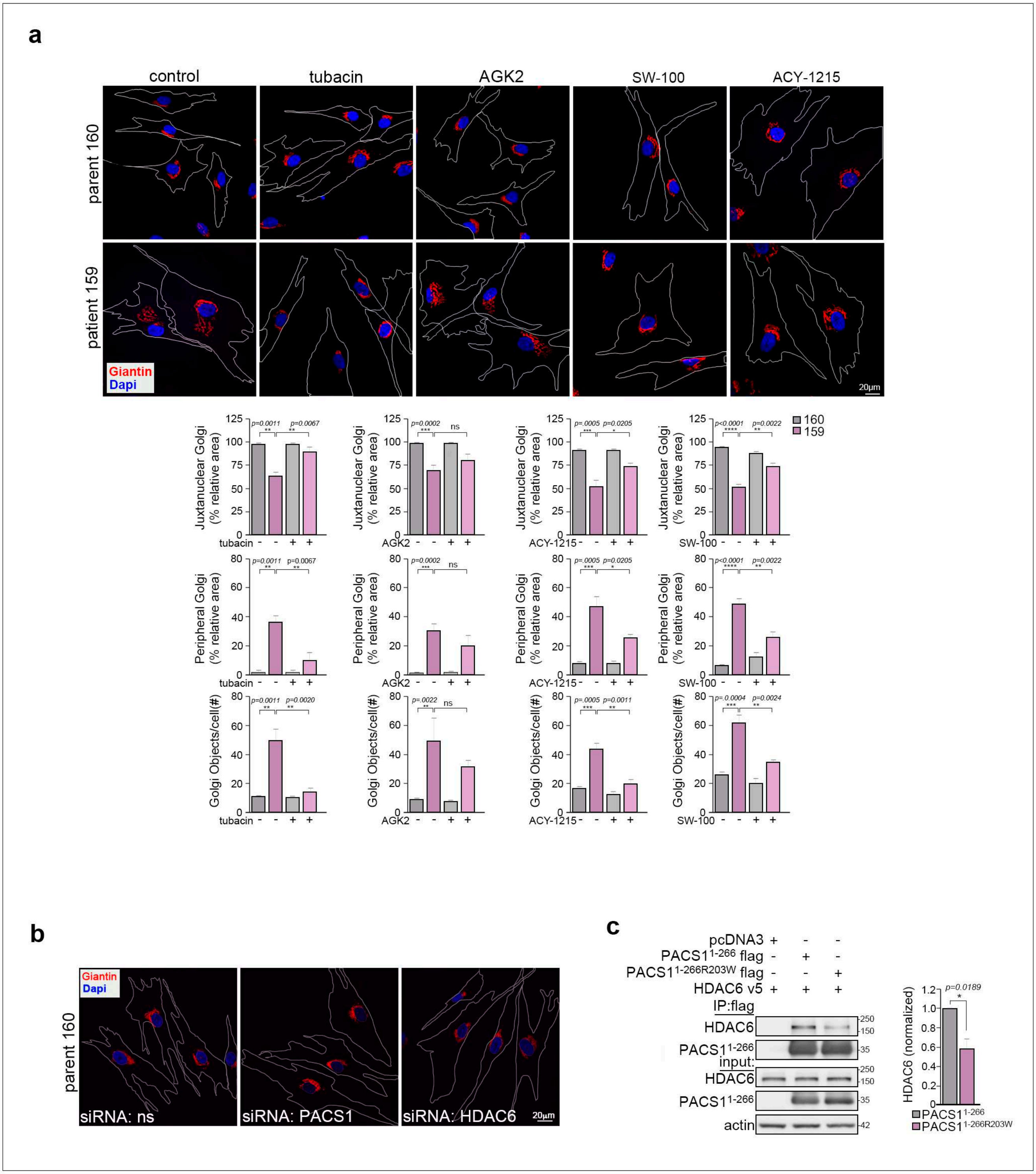
a
control
tubacin
AGK2
SW-100
ACY-1215
parent 160
patient 159
Giantin
Dapi
20µm
160
159
Juxtanuclear Golgi (% relative area)
Peripheral Golgi (% relative area)
Golgi Objects/cell(#)
tubacin
AGK2
ACY-1215
SW-100
b
parent 160
siRNA: ns
siRNA: PACS1
siRNA: HDAC6
c
pcDNA3
HDAC6 v5
IP:flag
HDAC6
input:
actin
HDAC6 (normalized)

Fig S2

Figure S3: (a) (Top) CRISPR-Cas9 methods were used to insert floxed cassettes designed to express HA-tagged PACS1 or $PACS1^{R203W}$ under control of the CAG promoter into the Rosa26 safe harbor locus. The IRES-dependent expression of the GFP sequence downstream of the PACS1 or $PACS1^{R203W}$ cDNAs was too low to be detected and was used only for PCR genotyping. (Bottom) PCR genotyping gel depicting the PCR primers used and the resultant PCR products that identify WT, $R26^{P1}$ and $R26^{P1R203W}$ lines. **(b)** IHC of coronal brain sections prepared from P11 *Emx1^{Cre};R26⁺*, *Emx1^{Cre};R26^{P1}* or *Emx1^{Cre};R26^{P1R203W}* female mice were stained for CTIP2 (green), SATB2 (red) and nuclei (DAPI). Scale bar, 250 µm. **(c)** Primary cultures of dissociated *Emx1^{Cre};R26^{P1}* and *Emx1^{Cre};R26^{P1R203W}* hippocampal neurons were fixed on DIV14 and stained for HA (green), Giantin (red) and nuclei (DAPI). Scale bar, 20 µm. Data are mean ± SEM, n=12 neurons/group. **(d)** WT C57BL/6 pups were injected (ICV) at P1 with a single dose of H6ASO or control nASO (40 µg) into one hemisphere. After 4 weeks, brains were harvested and the level of HDAC6 mRNA and protein were analyzed by qRT-PCR or Western blot, respectively. Data are mean ± SEM.

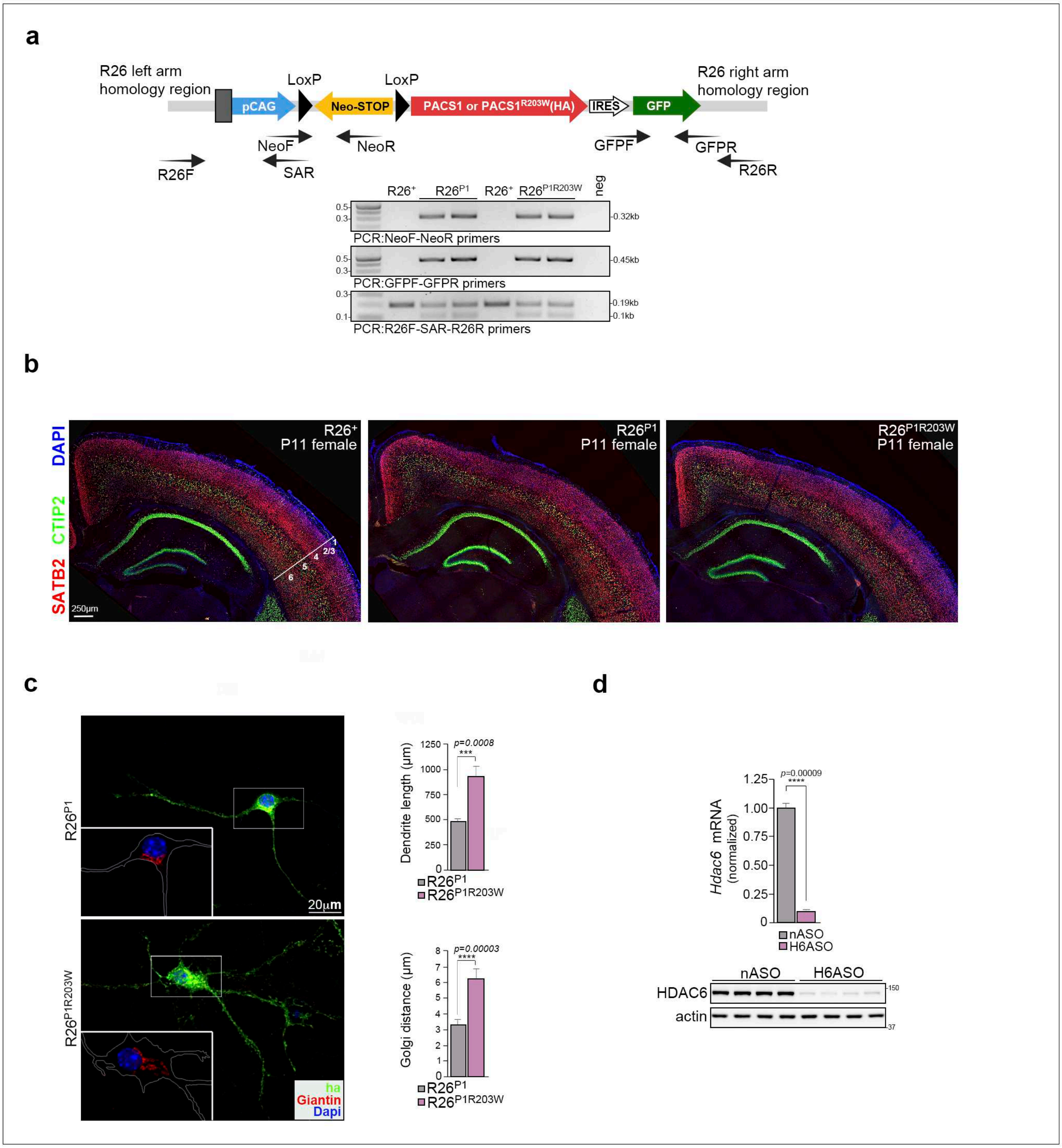
a
R26 left arm homology region
LoxP
LoxP
pCAG
Neo-STOP
PACS1 or PACS1R203W(HA)
IRES
GFP
R26 right arm homology region
NeoF
NeoR
R26F
SAR
GFPF
GFPR
R26R
R26+
R26P1
R26+
R26P1R203W
neg
0.32kb
PCR:NeoF-NeoR primers
0.45kb
PCR:GFPF-GFPR primers
0.19kb
0.1kb
PCR:R26F-SAR-R26R primers
b
DAPI
CTIP2
SATB2
R26+
P11 female
R26P1
P11 female
R26P1R203W
P11 female
250µm
c
R26P1
R26P1R203W
20µm
ha
Giantin
Dapi
p=0.0008
Dendrite length (µm)
p=0.00003
Golgi distance (µm)
d
p=0.00009
Hdac6 mRNA (normalized)
nASO
H6ASO
HDAC6
actin

Fig. S3

Figure S4: (a) Western blot of non-synaptosomal and synaptosomal fractions isolated from WT C57BL/6 forebrains. **(b)** (Left) Representative whole-cell voltage-clamp recordings of mIPSCs from L2/3 pyramidal neurons in acute brain slices of juvenile *Emx1*^{Cre}-induced R26^{P1} and R26^{P1R203W} mice injected (ICV) at P1 with 40 µg nASO or H6ASO. (Middle) mIPSC amplitude (top) and frequency (bottom) of R26^{P1} and R26^{P1R203W} mice injected at P1 with nASO or H6ASO. Data are mean ± SEM. (Right) Cumulative probability distributions of mIPSC amplitudes (top) and frequencies (bottom). n = 12-17 neurons/group, and 3-5 animals per condition (2-way ANOVA).

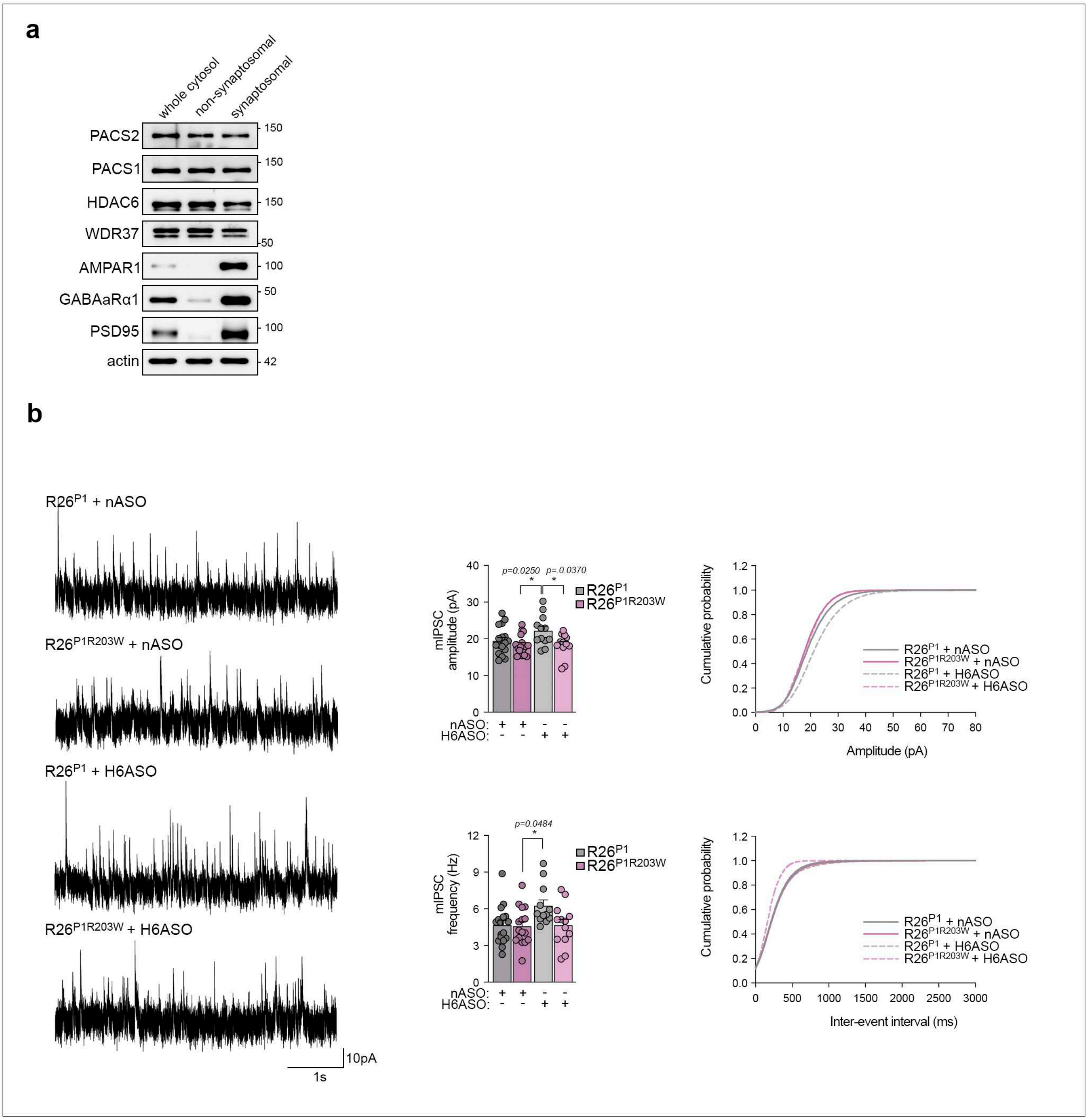
a
whole cytosol
non-synaptosomal
synaptosomal
PACS2
PACS1
HDAC6
WDR37
AMPAR1
GABAaRα1
PSD95
actin
150
150
150
50
100
50
100
42
b
R26P1 + nASO
R26P1R203W + nASO
R26P1 + H6ASO
R26P1R203W + H6ASO
10pA
1s
mIPSC amplitude (pA)
p=0.0250
p=0.0370
R26P1
R26P1R203W
nASO:
H6ASO:
Cumulative probability
Amplitude (pA)
mIPSC frequency (Hz)
p=0.0484
Inter-event interval (ms)

Fig S4

Figure S5: (a) (Top) Schematic of *Pacs1* exon 4 harboring the 4 bp deletion in the $Pacs1^{HET}$ mice, together with the location the PCR primers used for genotyping. (Bottom) PCR genotyping gel showing the PCR primers used and the resultant PCR products that identify $Pacs1^{WT}$, $Pacs1^{HET}$, and $Pacs1^{KO}$ mice. **(b)** Body mass of 6 wk old *WT*, $Pacs1^{HET}$ and $Pacs1^{KO}$ male and female littermates. Data are mean ± SEM, n=20-25/sex. **(c)** Western blot of Ac-cortactin, total cortactin and PACS1 in brain extracts prepared from adult $Pacs1^{WT}$ and $Pacs1^{KO}$ mice. **(d)** Western blot of total α-tubulin and $Ac\text{-}Lys^{40}$-α-tubulin in SV40-immortalized WT and $Pacs1^{KO}$ MEFs. Data are mean ± SD, n=3. **(e)** Western blot of total brain lysate prepared from WT and $Pacs1^{KO}$ mice. **(f)** (Left) Representative whole-cell voltage-clamp recordings of mEPSCs from L2/3 pyramidal neurons in acute brain slices of juvenile WT and $Pacs1^{KO}$ mice. (Middle) AMPAR mEPSC amplitude (top) and frequency (bottom) of WT and $Pacs1^{KO}$ mice. Data are mean ± SEM. (Right) Cumulative probability distributions of AMPAR mEPSC amplitudes (top) and frequencies (bottom) n = 10-12 neurons/group, and 3-4 animals per condition. **(g)** Representative whole-cell voltage-clamp recordings of mIPSCs from L2/3 pyramidal neurons in acute brain slices of juvenile WT and $Pacs1^{KO}$ mice. (Middle) mIPSC amplitude (top) and frequency (bottom) of juvenile WT and $Pacs1^{KO}$ mice. Data are mean ± SEM. (Right) Cumulative probability distributions of mIPSC amplitudes (top) and frequencies (bottom) n = 10-15 neurons/group, and 3-4 animals per condition.

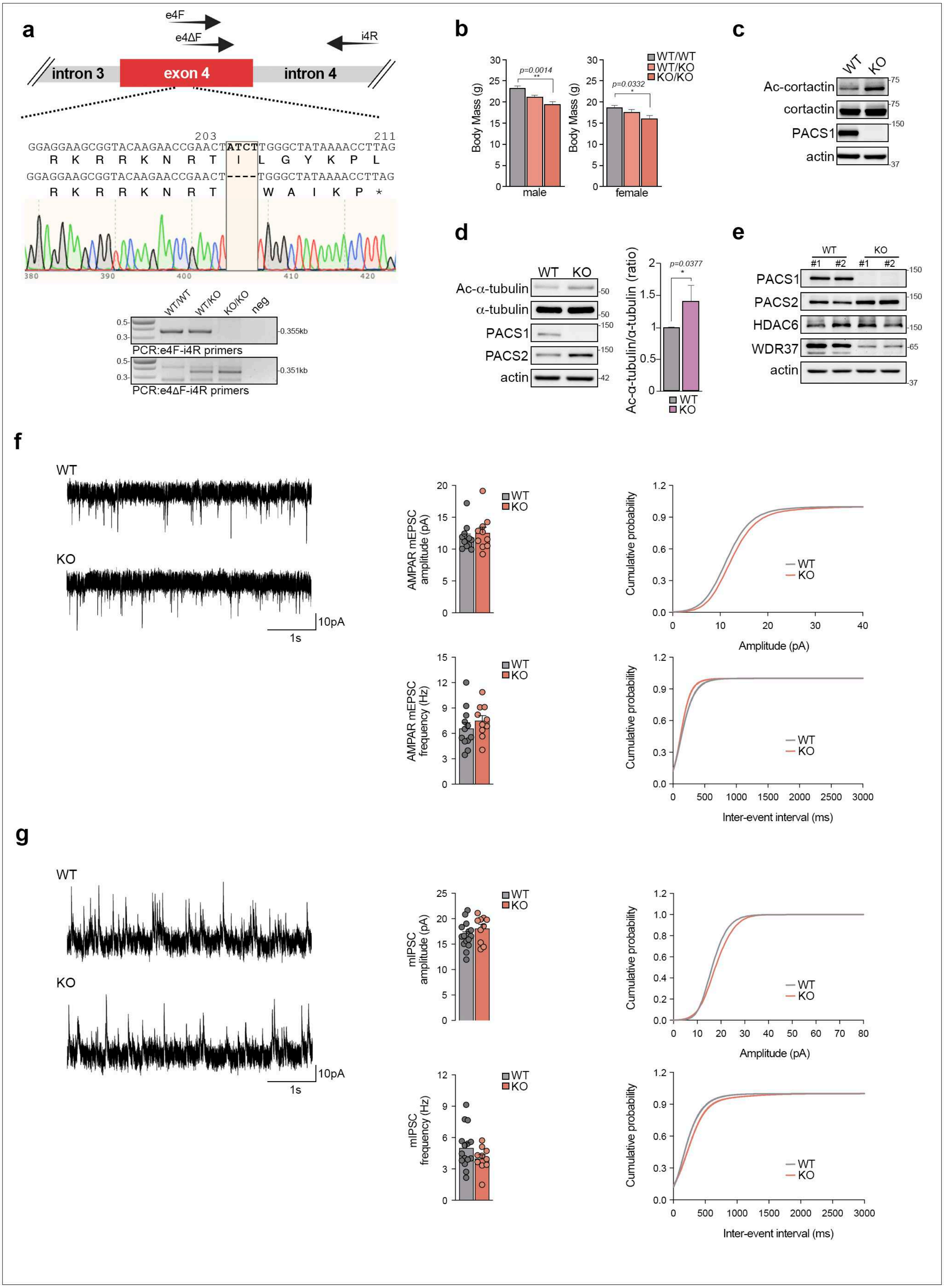
a
e4F
e4ΔF
i4R
intron 3
exon 4
intron 4
203
211
GGAGGAAGCGGTACAAGAACCGAACTATCTTGGGCTATAAAACCTTAG
R K R R K N R T I L G Y K P L
GGAGGAAGCGGTACAAGAACCGAACT----TGGGCTATAAAACCTTAG
R K R R K N R T W A I K P *
WT/WT
WT/KO
KO/KO
neg
PCR:e4F-i4R primers
PCR:e4ΔF-i4R primers
b
Body Mass (g)
male
female
p=0.0014
p=0.0332
WT/WT
WT/KO
KO/KO
c
WT
KO
Ac-cortactin
cortactin
PACS1
actin
d
Ac-α-tubulin
α-tubulin
PACS1
PACS2
actin
Ac-α-tubulin/α-tubulin (ratio)
p=0.0377
e
PACS1
PACS2
HDAC6
WDR37
actin
f
AMPAR mEPSC amplitude (pA)
AMPAR mEPSC frequency (Hz)
Cumulative probability
Amplitude (pA)
Inter-event interval (ms)
10pA
1s
g
mIPSC amplitude (pA)
mIPSC frequency (Hz)
Cumulative probability
Amplitude (pA)
Inter-event interval (ms)

Fig S5

Figure S6: (a) (Top) CRISPR-Cas9 methods were used to insert Megamer cassettes designed to conditionally express $PACS1^{R201W}$ or PACS1. (Bottom left) PCR genotyping gel depicting the PCR primers used and the resultant PCR products that identify $Pacs1^{M/+}$ and $Pacs1^{R201W/+}$ lines. (Bottom right) Western blot showing the $\mathit{Emx1}^{\mathit{Cre}}$ induction of the floxed *Pacs1* and $\mathit{Pacs1}^{\mathit{R201W}}$ alleles. **(b)** Representative whole-cell voltage-clamp recordings of mIPSCs from L2/3 pyramidal neurons in acute brain slices of juvenile $Pacs1^{M/+}$ and $Pacs1^{R201W/+}$ mice. (Middle) mIPSC amplitude (top) and frequency (bottom) of juvenile $Pacs1^{M/+}$ and $Pacs1^{R201W/+}$ mice. Data are mean ± SEM. (Right) Cumulative probability distributions of mIPSC amplitudes (top) and frequencies (bottom). n = 13-14 neurons/group, and 4-6 animals per condition (2-way ANOVA). **(c)** WT C57BL/6 pups were injected (ICV) at P1 with a single dose of P1ASO or control nASO (40 μg) into one hemisphere. After 4 weeks, brains were harvested and the level of *Pacs1* mRNA and protein were analyzed by qRT-PCR and Western blot. Data are mean ± SEM.

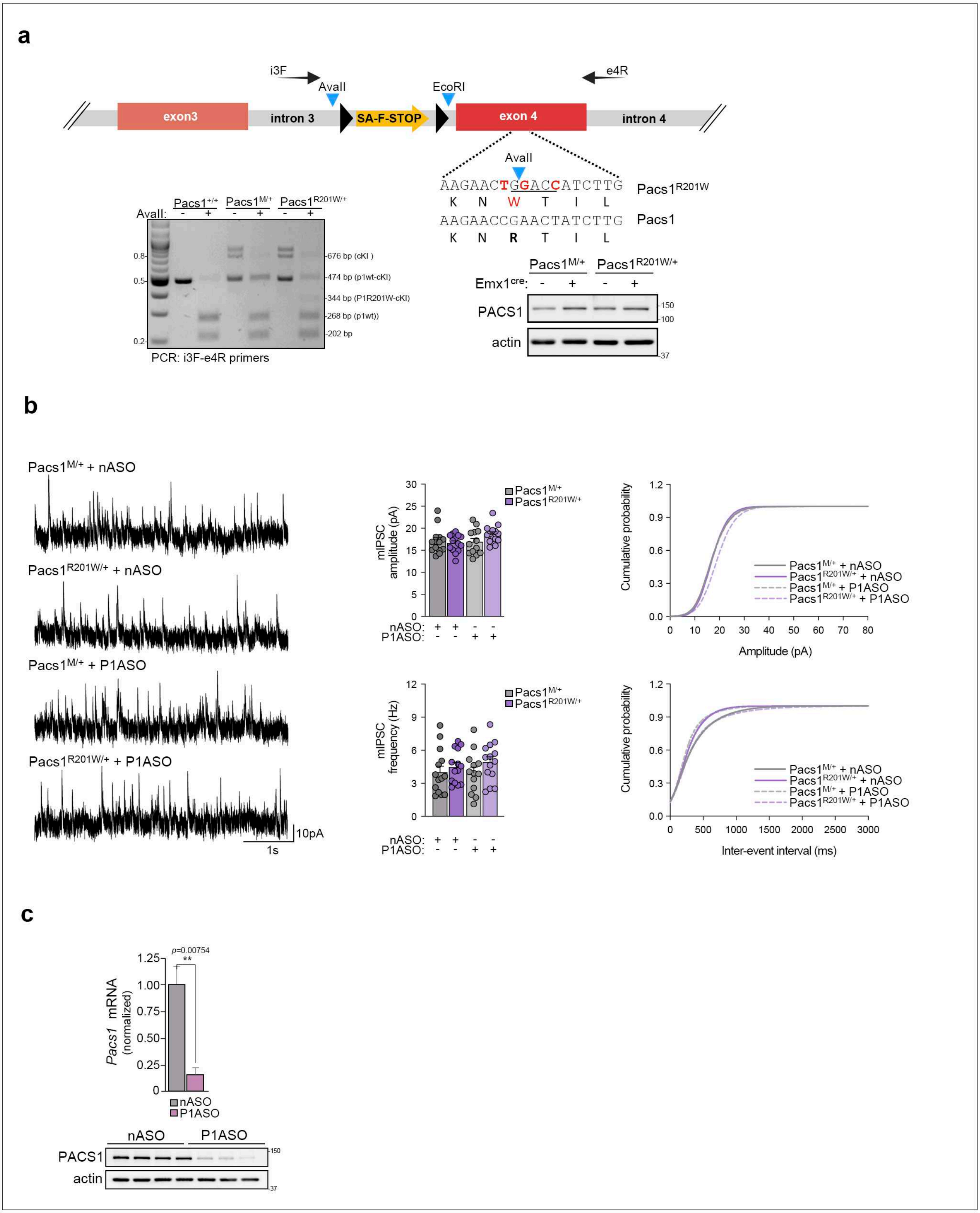
a
i3F
e4R
AvaII
EcoRI
exon3
intron 3
SA-F-STOP
exon 4
intron 4
AvaII
AAGAACTGGACCATCTTG
K N W T I L
Pacs1R201W
AAGAACCGAACTATCTTG
K N R T I L
Pacs1
Pacs1+/+
Pacs1M/+
Pacs1R201W/+
AvaII:
0.8
0.5
0.2
676 bp (cKI)
474 bp (p1wt-cKI)
344 bp (P1R201W-cKI)
268 bp (p1wt))
202 bp
PCR: i3F-e4R primers
Pacs1M/+
Pacs1R201W/+
Emx1cre:
PACS1
actin
150
100
37
b
Pacs1M/+ + nASO
Pacs1R201W/+ + nASO
Pacs1M/+ + P1ASO
Pacs1R201W/+ + P1ASO
10pA
1s
mIPSC amplitude (pA)
mIPSC frequency (Hz)
nASO:
P1ASO:
Cumulative probability
Amplitude (pA)
Inter-event interval (ms)
c
p=0.00754
**
Pacs1 mRNA (normalized)
nASO
P1ASO
PACS1
actin
150
37

Fig S6
